# Conjugation Dynamics of Self-Transmissible and Mobilisable Plasmids into *E. coli* O157:H7 on *Arabidopsis thaliana* Rosettes

**DOI:** 10.3390/antibiotics10080928

**Published:** 2021-07-30

**Authors:** Mitja N. P. Remus-Emsermann, David Aicher, Cosima Pelludat, Pascal Gisler, David Drissner

**Affiliations:** 1 Microbiology of Plant Foods, Agroscope, 8820 Waedenswil, Switzerland; pascal.gisler72@gmail.com; 2 School of Biological Sciences, University of Canterbury, Christchurch 8053, New Zealand; 3 Biomolecular Interaction Centre, University of Canterbury, Christchurch 8053, New Zealand; 4 Institute of Biology-Microbiology, Freie Universität Berlin, 14195 Berlin, Germany; 5 Department of Life Sciences, Albstadt-Sigmaringen University, 72488 Sigmaringen, Germany; aicher@hs-albsig.de; 6 Plant Pathology and Zoology in Fruit and Vegetable Production, Agroscope, 8820 Waedenswil, Switzerland; cosima.pelludat@agroscope.admin.ch

**Keywords:** antibiotic resistance, conjugation, plasmids, phyllosphere

## Abstract

Many antibiotic resistance genes present in human pathogenic bacteria are believed to originate from environmental bacteria. Conjugation of antibiotic resistance conferring plasmids is considered to be one of the major reasons for the increasing prevalence of antibiotic resistances. A hotspot for plasmid-based horizontal gene transfer is the phyllosphere, i.e., the surfaces of aboveground plant parts. Bacteria in the phyllosphere might serve as intermediate hosts with transfer capability to human pathogenic bacteria. In this study, the exchange of mobilisable and self-transmissible plasmids via conjugation was evaluated. The conjugation from the laboratory strain *Escherichia coli* S17-1, the model phyllosphere coloniser *Pantoea eucalypti* 299R, and the model pathogen *E. coli* O157:H7 to the recipient strain *E. coli* O157:H7::MRE103 (*Ec*O157:H7red) in the phyllosphere of *Arabidopsis thaliana* was determined. The results suggest that short-term occurrence of a competent donor is sufficient to fix plasmids in a recipient population of *E. coli* O157:H7red. The spread of self-transmissible plasmids was limited after initial steep increases of transconjugants that contributed up to 10% of the total recipient population. The here-presented data of plasmid transfer will be important for future modelling approaches to estimate environmental spread of antibiotic resistance in agricultural production environments.

## 1. Introduction

With the introduction of penicillin in the 1940s, mankind entered the era of antibiotics (AB), which revolutionised therapeutic medicine [1,2]. For the first time, physicians were able to cure their patients of deadly bacterial diseases and saved millions of lives [3]. Less than a century later, bacterial diseases have yet again become a major threat to human welfare as infectious bacteria acquired antibiotic resistances (ABR) that are able to overcome every antibiotic currently available [3,4]. ABR per se is a natural phenomenon in bacteria [5] and its main function is likely a countermeasure against antibiotic-producing microorganisms that compete for the same resources [6]. It is the use of AB in anthropogenic applications such as medical treatment, animal husbandry, and agricultural practice that spreads ABR in environmental and infectious bacteria whilst pushing the selection pressure on a level beyond the natural evolutionary clock [7].

Many ABR genes present in human pathogenic bacteria are believed to originate from environmental bacteria [8,9,10,11]. This implies that, for an ABR gene to reach a human pathogenic bacterium, there needs to be an exchange of genetic material from environmental bacteria towards pathogens. Transfer of genetic material can be achieved by uptake of environmental DNA due to natural competence, phage-mediated transduction, integrative and conjugative elements, or conjugation of plasmids [12,13]. The latter is considered to be a major cause for the increased prevalence of ABR [8,9]. The ability of conjugative plasmids to move genes from one bacterium to another, not necessarily related to each other, is responsible for the rapid spread and accumulation of resistances [9,14,15,16]. A hotspot for plasmid-based horizontal gene transfer is the phyllosphere [17,18,19,20,21], representing the surface of all above-ground organs of land plants [22], thereby including the fresh plant products that are considered an important part of a healthy diet.

In today’s intensive agricultural production, fertilisers are needed to replenish soil nutrients, such as nitrogen and phosphorus. They are essential for crop growth and increased crop yield. Animal manure is an excellent source for such nutrients, but it often originates from intensive animal husbandry farms, where the widespread use of AB to preventively treat animals is the rule rather than the exception [23]. This leads not only to a relative increase of ABR bacteria in faecal waste, but also to an accumulation of ABR-conferring genetic elements, such as plasmids [23,24,25]. Bacteria that constitute the normal phyllosphere microbiota are generally not considered harmful [26,27], but they might serve as intermediate hosts for ABR-conferring plasmids with transfer capability to human pathogenic bacteria. Little is known about the number of transfer steps involved in the propagation of resistance genes and the efficacy of the mechanism participating in the exchange of genetic material in the environment. However, information about plasmid transfer and plasmid persistence will be important for future modelling and risk assessment approaches to estimate environmental spread of antibiotic resistance in agricultural production environments.

**Figure 1:**
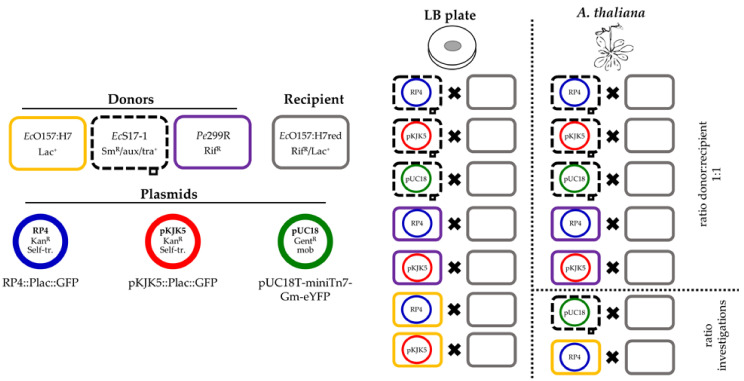
Overview of performed experiments. Three donors, *Ec*O157:H7, *Ec*S17-1, and *Pe*299R, were combined with up to three different plasmids, RP4, pKJK5, or pUC18. The ability of the hosts to transfer the plasmids to the recipient *Ec*O157:H7red was assessed on membrane filters and gnotobiotic *A. thaliana*. Additionally, the effect of different donor/recipient ratios onto conjugation frequency was assessed for two exemplary donor and plasmid combinations.

In the present study, a laboratory-scale model system was established that mimics the shortest possible route for ABR-carrying plasmids into pathogenic *Escherichia coli* O157:H7::MRE103 Δ*stx* (*Ec*O157:H7red) recipients on *Arabidopsis thaliana* rosettes. The exchange of the mobilisable plasmid pUC18T-mini-Tn7T-Gm-eYFP (pUC18) and self-transmissible ABR-carrying plasmids pKJK5::Plac:gfp (pKJK5) and RP4::Plac:gfp (RP4) via conjugation by filter mating and in the phyllosphere of *A. thaliana* was evaluated. Donors are either the model phyllosphere colonising strain *Pantoea eucalypti* 299R (*Pe*299R), the non-pathogenic laboratory strain *E. coli* S17-1 (*Ec*S17-1), or *E. coli* O157:H7 Δ*stx* (*Ec*O157:H7) (Figure 1). The assay takes into account that plants can carry human pathogenic bacteria as contaminations, originating from animal manure or the digestates from biogas plants, which are used as organic fertiliser [21,28,29]. Organic fertilisers are also sources for ABR-conferring genetic elements, such as plasmids [24,25]. To mimic natural conditions, we conducted in planta experiments in the absence of antibiotic pressure.

## 2. Results

### 2.1. Transconjugant Frequencies after Filter Mating

**Figure 2:**
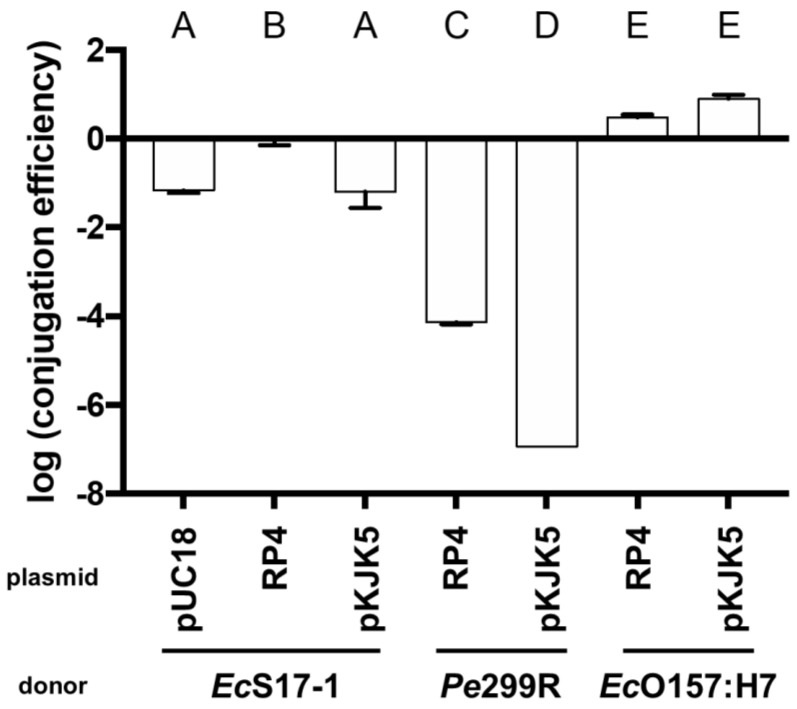
Transconjugant frequencies in the recipient population after mating of the recipient *Ec*O157:H7red with *Ec*S17-1, *Pe*299R, and *Ec*O157:H7 donors carrying plasmids pUC18, pKJK5, or RP4 on nitrocellulose filters. Error bars represent the standard deviation of the mean. Significant differences between treatments are marked with different letters (*p*-value < 0.01, one-way ANOVA, Tukey’s multiple comparison test).

Matings on nitrocellulose filters were performed to determine transconjugant frequencies of the recipient *Ec*O157:H7red (Figure 2). Besides *Pe*299R (pKJK5), all donors were able to transfer plasmids to *Ec*O157:H7red. In the case of *Ec*S17-1 being the donor, all plasmids were transferred at high rates, and the transconjugant frequency was between 10^0^ and 6.31 × 10^−2^ per recipient cell depending on the transmitted plasmid (transconjugant frequencies pKJK5 < pUC18 < RP4). When *Pe*299R was donor of RP4 or pKJK5, transconjugants were on average detected at frequencies of 6.82 × 10^−5^ or below the limit of detection, which was 9.62 × 10^−6^ per recipient cell, respectively. *Ec*O157:H7 donors transferred the plasmids pKJK5 and RP4 with the highest efficiency to *Ec*O157:H7red with transconjugants being detected at frequencies of 3.16 × 10^1^ and 7.94 × 10^1^ (transconjugant frequencies RP4 < pKJK5). Since, in contrast to *Ec*S17-1, *Pe*299R and *Ec*O157:H7 are not able to mobilise plasmids, they were not tested as donors of pUC18.

### 2.2. Growth Dynamics of Individual or Co-Inoculated Bacterial Strains in Planta

**Figure 3:**
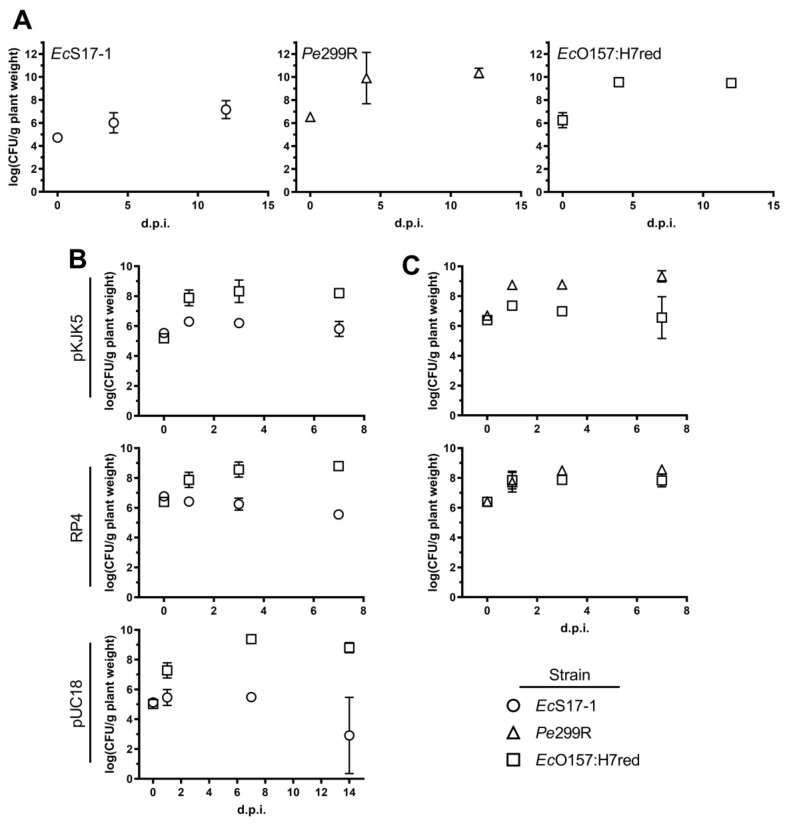
Bacterial population development on gnotobiotic *A. thaliana.* (**A**) Populations after monoinoculation of *Ec*S17-1 (circles), *Pe*299R (triangles), or *Ec*O157:H7red (squares) at 4 and 12 days post-inoculation (d.p.i.). (**B**) Population developments after co-inoculation of *Ec*S17-1 (circles) harbouring pKJK5 (top), RP4 (middle), and pUC18 (bottom) with the recipient *Ec*O157:H7red (squares) onto gnotobiotic *A. thaliana* after 1, 3, and 7 d.p.i. (**C**) Population development after co-inoculation of *Pe*299R (triangles) harbouring pKJK5 (top) and RP4 (bottom) with the recipient *Ec*O157:H7red (squares) onto gnotobiotic *A. thaliana* after 1, 3, and 7 d.p.i. Error bars represent the standard deviation of the mean.

To determine the ability of the different strains to colonise *A. thaliana*, we inoculated *Ec*S17-1, *Pe*299R, and *Ec*O157:H7red (representative also for donor *Ec*O157:H7) onto axenic plants. When grown individually, all bacterial strains, including the auxotrophic laboratory strain *Ec*S17-1, were able to grow to high densities on *A. thaliana*, reaching CFU counts of 10^8^−10^10^ bacteria per gram plant material after 4 and 12 days post-inoculation (d.p.i.) (Figure 3A); similar densities of bacterial colonisation have been reported before in gnotobiotic and environmental studies [30,31]. When *Ec*S17-1 or *Pe*299R carrying either self-transmissible plasmids RP4 or pKJK5 were co-inoculated with *Ec*O157:H7red, population development of individual strains behaved differently (Figure 3B,C). When co-cultured with *Ec*S17-1 (pKJK5), the *Ec*O157:H7red population reached similar densities as grown on *A. thaliana* only, i.e.*, Ec*O157:H7red multiplied to densities of >10^8^ CFU g^−1^ and maintained those densities until the end of the experiment. In contrast, *Ec*S17-1 (pKJK5) increased to a maximum of >10^6^ CFU g^−1^ after 24 h, but then the population steadily declined below 10^6^ CFU g^−1^ until 7 d.p.i. (Figure 3B, top). When *Ec*O157:H7red was co-cultured with *Ec*S17-1 (RP4), the initial population size of *Ec*S17-1 (RP4) was 10^7^ CFU g^−1^ and the population did not further increase and steadily declined during the experiment (Figure 3B, middle). The *Ec*O157:H7red population increased by two magnitudes to 5 × 10^8^ CFU g^−1^ and remained stable. When in co-culture with *Ec*S17-1 (pUC18), *Ec*O157:H7red showed a strong initial increase and reached a maximum density of >10^9^ CFU g^−1^, 7 days after inoculation (Figure 3B, bottom). *Ec*S17-1 (pUC18) reached a maximum density of ≈10^5^ CFU g^−1^, 1 day after inoculation. Afterwards, the density remained stable and started declining 7 days after inoculation.

When in competition with *Pe*299R (pKJK5), the *Ec*O157:H7 population increased by one magnitude to 10^7^ CFU g^−1^ and never reached densities of above 10^8^ CFU g^−1^, while *Pe*299R (pKJK5) increased from 10^6^ CFU g^−1^ to 10^9^ and remained stable for the duration of the experiment (Figure 3C, top). In competition with *Pe*299R (RP4), both *Ec*O157:H7red and *Pe*299R (RP4) reached densities of approximately 10^8^ CFU g^−1^ and remained stable thereafter (Figure 3C, bottom).

When *Ec*O157:H7 represented the donor, the combined *Ec*O157:H7 population (sum of donor and recipient CFU) reached cell numbers above 10^8^ CFU g^−1^ (Figure S1).

### 2.3. Conjugation Dynamics of Self-Transmissible Plasmids in Planta

**Figure 4:**
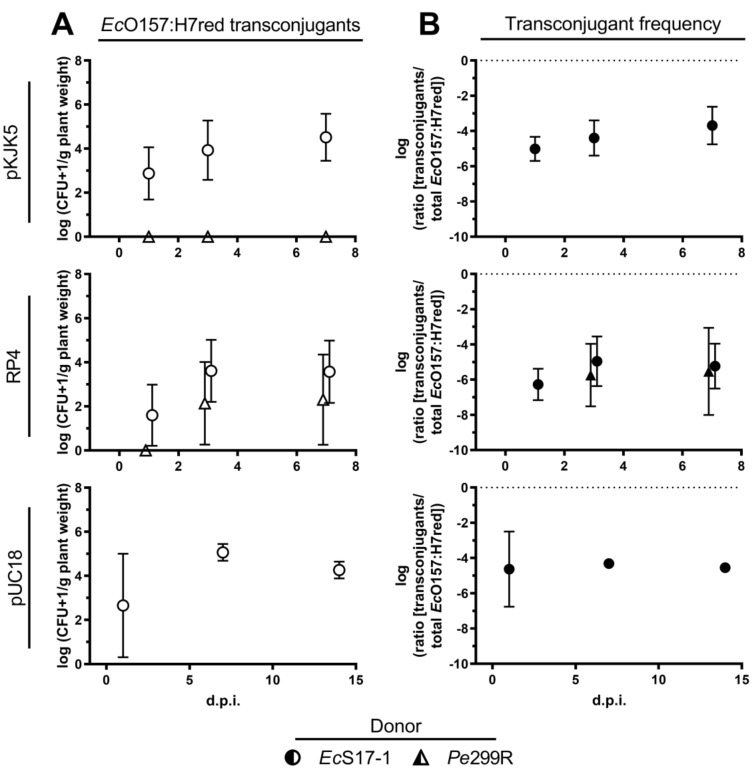
Conjugation dynamics of transconjugant *Ec*O157:H7red on gnotobiotic *A. thaliana*. (**A**) Population development of transconjugant *Ec*O157:H7red after co-inoculation with donors *Ec*S17-1 (circles) and *Pe*299R (triangles) harbouring pKJK5 (top), RP4 (middle), and pUC18 (bottom) onto gnotobiotic *A. thaliana* after 1, 3, and 7 d.p.i. (**B**) Frequency of *Ec*O157:H7red transconjugants in the recipient population after co-inoculation with donors *Ec*S17-1 (circles) and *Pe*299R (triangles) harbouring pKJK5 (top), RP4 (middle), and pUC18 (bottom) onto gnotobiotic *A. thaliana* after 1, 3, and 7 d.p.i. The conjugation with *Pe*299R (pKJK5) did not yield transconjugants above the limit of detection. Error bars represent the standard deviation of the mean.

*Ec*S17-1 was able to transfer self-transmissible pKJK5 and RP4 to *Ec*O157:H7red on *A. thaliana*. When co-inoculated for 24 h with *Ec*S17-1 (pKJK5) or *Ec*S17-1 (RP4), on average, more than 10^3^ *Ec*O157:H7red (pKJK5) and ≈10^2^ *Ec*O157:H7red (RP4) transconjugants g^−1^ plant were detected (Figure 4A, top and middle). The average ratio of *Ec*O157:H7red transconjugants in the recipient population slowly increased over time, but not significantly (Figure 4B, top and middle).

No transconjugants could be detected after co-inoculation of *Pe*299R (pKJK5) and *Ec*O157:H7red. After co-inoculation of *Pe*299R (RP4) and *Ec*O157:H7red, 5 × 10^2^ *Ec*O157:H7red transconjugants were detected 7 days after inoculation (Figure 4A, middle). Between replicates, the occurrence of transconjugants was highly fluctuating, resulting in large standard deviations. The frequency of transconjugants remained stable during the period of 7 days (Figure 4B, middle).

### 2.4. Effect of Non-Self-Transmissible but Mobilisable Plasmids on Transconjugant Frequencies in Planta

*Ec*S17-1 was able to transfer the mobilisable plasmid pUC18 to *Ec*O157:H7red on *A. thaliana*. When co-inoculated for 24 h with *Ec*S17-1 (pUC18), on average, 10^3^ *Ec*O157:H7red (pUC18) transconjugants g^−1^ plant were detected, reaching a maximum of ≈10^5^ transconjugants g^−1^ plant 7 d.p.i. (Figure 4A, bottom). The average frequency of *Ec*O157:H7red (pUC18) transconjugants reached 10^−4^ per recipient cell after 7 days and did not decrease within 14 days, despite the lack of selective agents (i.e., antibiotics) and a potential fitness cost of the plasmid (Figure 4B, bottom).

### 2.5. Effect of Ratio Differences between Donor Cells to Recipients on Transconjugant Frequencies in Planta

**Figure 5:**
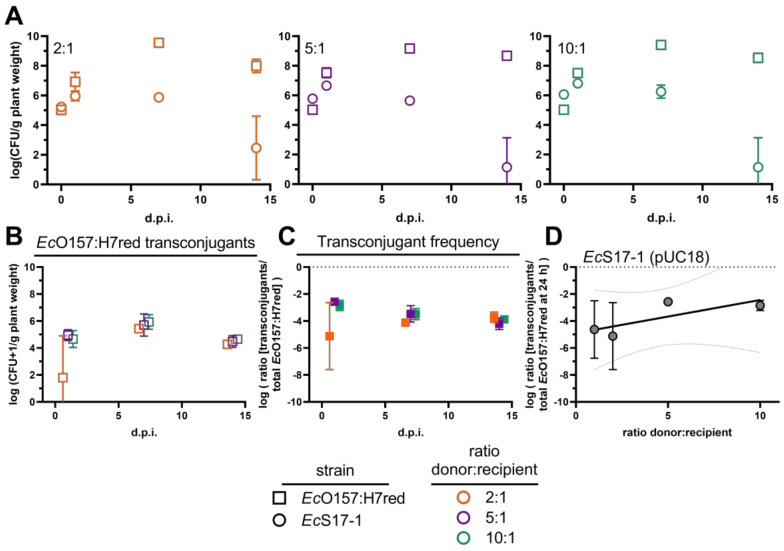
Conjugation dynamics of the mobilisable plasmid pUC18 after inoculating different densities of donors. (**A**) Population developments after co-inoculation of different ratios of the donor *Ec*S17-1 (circles) harbouring pUC18 with the recipient *Ec*O157:H7red (squares) onto gnotobiotic *A. thaliana* after 1, 7, and 14 d.p.i. The initial recipient inoculation density remained constant, whilst *Ec*S17-1 donors were inoculated at different densities, resulting in donor/recipient ratios of 2:1 (left, orange), 5:1 (middle, purple), and 10:1 (right, green). (**B**) Population development of transconjugant *Ec*O157:H7red after co-inoculation with different densities of donor *Ec*S17-1 (pUC18) onto gnotobiotic *A. thaliana* after 1, 7, and 14 d.p.i. (**C**) Frequency of *Ec*O157:H7red transconjugants in the recipient population after co-inoculation with different densities of donor *Ec*S17-1 (pUC18) onto gnotobiotic *A. thaliana* after 1, 7, and 14 d.p.i. (**D**) Transconjugant frequencies of different donor/recipient ratios after 24 h. No significant differences in the conjugation efficiency after treatments with different *Ec*S17-1 donor concentrations could be detected, however, the variation within treatments was lower at high donor densities. A linear regression was fitted (r^2^ = 0.61, broken lines 95% confidence intervals of the linear regression). Error bars represent the standard deviation of the mean.

To separate the effect of secondary horizontal transfer of plasmids from primary conjugations, i.e., from a freshly conjugated cell to another recipient vs. from an original donor to a recipient cell, we tested three different initial densities of donor *Ec*S17-1 carrying the mobilisable, but non-self-transmissible, plasmid pUC18 and *Ec*O157:H7red as recipient. Donor and recipient were mixed in ratios of 1:1 (see Section 2.4), 2:1, 5:1, and 10:1. Similar to the same starting densities of donor and recipient, *Ec*O157:H7red showed a strong initial increase and reached a maximum density of >10^9^ CFU g^−1^, 7 days after inoculation for all donor/recipient ratios (Figure 5A). *Ec*S17-1 (pUC18) reached a maximum density at day 1 after inoculation and then started declining. The donor was outcompeted by the recipient during the experiment (Figure 5A). Additionally, a strong initial increase of *Ec*O157:H7red transconjugants occurred within the first 24 h for all of the different donor/recipient ratios and remained stable until the end of the experiment (Figure 5B). Interestingly, while not statistically significant, all analysed timepoints showed a trend of higher transconjugant numbers in the presence of increased donor densities (Figure 5B). As a consequence of the competition between donor cells and recipient cells, the probability over time for recipients to encounter donor cells decreased, which was supported by a slight decline of transconjugant frequencies over the course of time (Figure 5C).

When comparing the transconjugant frequencies after treatment with different donor densities 24 h after inoculation, we found no significant difference between the different donor and recipient ratios. However, we found a positive trend between donor density and transconjugant frequencies after 24 h (Figure 5D).

To investigate the effect of donor to recipient ratios onto the ability of self-transmissible plasmids to invade a population of *E. coli* O157:H7, *Ec*O157:H7 (RP4) donor, and *Ec*O157:H7red, we inoculated recipient ratios 1:10, 1:5, 1:2, 2:1, 5:1, and 10:1 onto *A. thaliana* plants. All ratio mixtures yielded >10^4^ transconjugants per gram of plant after 24 h, and the transconjugant population levelled off between 10^6^ and 10^7^ transconjugants per gram of plant (Figure S1A). This translated to transconjugant frequencies of 2.5 × 10^−2^ to 9 × 10^−4^ for the different ratio mixtures 24 h after inoculation (Figure S1B).

**Figure 6:**
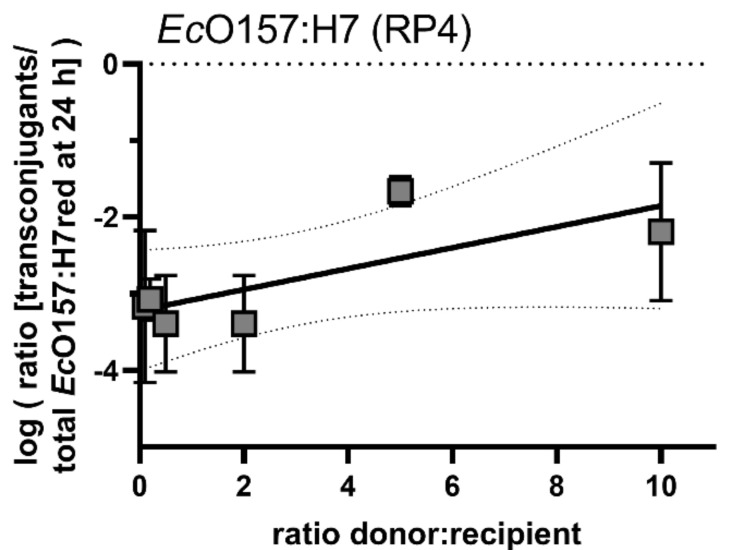
Conjugation dynamics of the self-transmissible plasmid RP4 after inoculating different densities of donor *Ec*O157:H7 with the recipient *Ec*O157:H7red. Shown are transconjugant frequencies of different donor/recipient ratios after 24 h. No significant differences in the conjugation efficiency after treatments with different *Ec*O157:H7 donor concentrations could be detected. A linear regression was fitted (r^2^ = 0.55, broken lines 95% confidence intervals of the linear regression). Error bars represent the standard deviation of the mean.

Similar to the results with mobilisable plasmid pUC18, transconjugant frequency data suggest a low correlation between the donor/recipient ratio and the transconjugant frequency after 24 h (Figure 6).

## 3. Discussion

To study the probability of horizontal gene transfer towards pathogenic bacteria on plant leaf surfaces, we established a model system for the exchange of self-transmissible- and non-self-transmissible but mobilisable plasmids. The model system provided insights into the conjugation between Enterobacteriaceae in the phyllosphere of *A. thaliana*. Besides the phyllosphere colonising strain *Pe*299R (pKJK5), all donor strains tested were able to transfer plasmids in measurable rates to the model human pathogenic *Ec*O157:H7red after being co-inoculated onto nitrocellulose filters, although *Pe*299R did so at a much lower frequency compared to *Ec*S17-1. The reason for this transfer barrier [12,32] is currently unknown, given that *Pe*299R was a competent recipient of the self-transmissible plasmid, that *Ec*O157:H7red had no issue with receiving the same plasmids from *Ec*S17-1, and that both donor and recipient are members of the family Enterobacteriaceae.

In planta, *Ec*O157:H7red outgrew *Ec*S17-1 and thereby outcompeted it. This is not unexpected, since both strains of *E. coli* should have a close to identical resource demand and *Ec*S17-1 is an auxotroph, lab-adapted strain [33], which is therefore prone to be less competitive. When co-inoculated with the phyllosphere-competent strain *Pe*299R carrying plasmid pKJK5, *Ec*O157:H7red did not reach the same high densities as in a monoculture, and the cell density was decreased to less than 10^7^ CFU on average. Potentially, *Pe*299R is outcompeting *Ec*O157:H7red due to nutritional competition [34]. There is no indication that *Pe*299R produces antibiotics which inhibit the growth of *Ec*O157:H7red [35] as no antibiotic production genes are annotated in the *Pe*299R genome [36] and no growth halos were formed around *Pe*299R colonies on agar plates indicative for growth inhibition of *Ec*O157:H7red (data not shown).

After co-inoculation of *Ec*S17-1 containing different self-transmissible and mobilisable plasmids with *Ec*O157:H7red as recipient, transconjugants could be detected after 24 h (Figure 4) at high rates, underlining the donor’s ability to transfer plasmids on plant leaves. These rates are comparable to previously reported conjugation rates in planta [18,19,37].

The physicochemical nature of plant leaf surfaces presents a spatially segregated, heterogeneous environment [38] that promotes clonal cluster formation and limits movement, thereby limiting the potential spread of invasive plasmids [39,40]. This might explain why self-transmissible plasmids did not further invade the recipient population and the relative contribution of plasmid-bearing transconjugants did not over-proportionally increase in time (Figure 4).

The extent to which the self-transmissible plasmid RP4 is able to invade the recipient population was tested by using *Ec*O157:H7 as donor and *Ec*O157:H7red as recipient. After an initial steep increase of the emerging transconjugant population within the first 24 h, the transconjugant population’s increase exhibited a slope that was slightly higher than the overall recipient’s population increase. This indicates that the plasmid was horizontally propagating to new recipients and the increase in the population not exclusively vertically during bacterial growth. Generally, after three days of growth, the increase in transconjugants levelled off and the contribution of transconjugants to the total *Ec*O157:H7red population did not further increase. This indicates that the ability of plasmids to invade the complete population is limited and directly connected to active growth of the donor and recipient populations. Once the plant is saturated with colonisers, the transmission of the plasmid slows to a hold and can best be explained by vertical transmission rather than horizontal transmission. By using a wide range of donor vs. recipient ratios that were initially inoculated, we determined the relationship between donor and recipient ratios and transconjugant frequencies. The transconjugant frequency was correlated with the number of donors inoculated (r^2^ = 0.55, Figure 6). The transconjugants frequency is likely a combined result of the maximal load of local leaf habitats [39] and the probability of members of the two populations to colonise the leaf at the same site [40,41].

When the non-self-transmissible but mobilisable plasmid pUC18 is conjugated by *Ec*S17-1, the transconjugant population is not over-proportionally increasing in comparison to self-transmissible plasmids. This lack of increase is likely depicting a stable total population of transconjugants that ceased in growth. As pUC18 does not contain the transfer machinery necessary to further conjugate itself, *Ec*O157:H7red transconjugants are incapable of transmitting the acquired plasmid to other cells. As expected, the ability of pUC18 to invade the recipient population is limited and the transconjugant population is increasing proportionally slower than the total recipient population. As the generation of new transconjugants is limited by the presence of the donor strain and vertical transfer of the plasmid from primary transconjugants to daughter cells, this can be interpreted as a cease of growth or decrease of the donor population and a cease of growth of the primary transconjugant population. Indeed, the donor population stopped growing after 1 day and started to decrease after 7 days (Figure 3B).

In line with previous findings, we observed that conjugation efficiency of plasmids was high in the absence of antibiotic pressure [42]. Even for mobilisable plasmids, which only propagate vertically after the initial conjugation, we found that transconjugants were not lost from the system, i.e., they were not outcompeted by the non-plasmid-bearing population. This finding is concerning as it indicates that even low frequencies of plasmid transfer on plant foodstuffs might fix a plasmid bearing antibiotic resistance in a population of bacteria.

## 4. Materials and Methods

### 4.1. Bacterial Strains and Growth Conditions

Strains and plasmids used in this study and their abbreviations are listed in Table 1. *E. coli* strains and *Pe*299R were routinely grown on lysogeny broth agar (LB). To determine total colony forming units (CFU) of *E. coli* after conjugation experiments, M9 minimal medium agar containing lactose as sole carbon source (15 g L^−1^ agar, 100 mL 10 × M9 salts (85.1 g L^−1^ Na_2_HPO_4_·2H_2_O, 30 g L^−1^ KH_2_PO_4_, 5 g L^−1^ NaCl, and 10 g L^−1^ NH_4_Cl, pH 7), 2 mL 1 M MgSO_4_, 1 mL 0.1 M CaCl, 40 mL 10% w/v lactose solution) or LB supplemented with rifampicin were employed. *E. coli* colonies were assessed after 7 days of incubation at room temperature, *Pe*299R colonies on the same agar plates after additional 7 days of incubation. To select for *Ec*O157:H7red transconjugants, we employed M9 minimal medium agar containing lactose as sole carbon source and appropriate antibiotics. *Ec*S17-1 CFU were determined by plating on LB agar containing streptomycin. To select for *Ec*O157:H7 (RP4) donor cells, we used LB containing kanamycin (transconjugants contributed to less than 10% of the donor population that was also kanamycin-resistant). Where appropriate, antibiotics were used in the following concentrations: kanamycin 50 µg mL^−1^, gentamicin 15 µg mL^−1^, streptomycin 100 µg mL^−1^, and rifampicin 100 µg mL^−1^.

### 4.2. Plasmids Used in the Study

The plasmids employed in this study (Table 1) are the two self-transmissible plasmids RP4::Plac::GFP (RP4), pKJK5::Plac::GFP (pKJK5) [15], and the mobilisable plasmid pUC18T-mini-Tn7T-Gm-eYFP (pUC18) [43]. Both self-transmissible plasmids are promiscuous and have a broad host range, RP4 is a IncP-1α incompatibility group plasmid [44] and pKJK5 is an IncP-1ε incompatibility group plasmid [45]. Plasmid pUC18 is a synthetic construct replicating only in Enterobacteriaceae and is present in high copy numbers when carried by *E. coli* [43]. For horizontal transfer plasmid pUC18 requires the *tra* operon, which *Ec*S17-1 contains in its chromosome.

### 4.3. Conjugation Experiments

An overview of the performed conjugation experiments is shown in Figure 1.

#### 4.3.1. Conjugation on Nitrocellulose Filters

To determine in vitro conjugation rates, we grew donors and recipients as described above. To prepare conjugation mixtures, we harvested a loop-full of cell material from freshly grown bacterial lawns on agar plates. Each individual strain was resuspended in 1 mL 1 × PBS (8 g L^−1^ NaCl, 0.24 g L^−1^ KCl, 1.42 g L^−1^ Na_2_HPO_4_, 0.24 g L^−1^ KH_2_PO_4_) by vortexing and pipetting, washed twice by centrifugation at 3500× *g*, and resuspended in 10 mL 1 × PBS. Optical density at 600 nm was determined for the cell suspensions, and 10^9^ CFU of donors and recipients, i.e., 1 mL of OD_600nm_ = 1 were mixed and concentrated by centrifugation. The mixtures were resuspended in 100 µL 1 × PBS, pipetted onto a nitrocellulose filter (0.22 µm pore diameter, Millipore, Burlington, MA, USA), placed on top of LB agar plates, and incubated at 30 °C. Bacteria were harvested after 24 h by placing the filter in an Eppendorf vial containing 1 mL 1 × PBS. The vial was vortexed until the complete bacterial biomass was dislodged and resuspended. From this suspension, a serial dilution was prepared up to 10^−11^, and 3 µL droplets were plated onto M9 lactose agar containing appropriate antibiotics to select for transconjugant *Ec*O157:H7red. Conjugation frequency was calculated by the ratio of *Ec*H157:H7red transconjugants to the total *Ec*O157:H7red population. Conjugation data are known to be log-normal distributed; thus, transconjugant and donor CFU numbers were log_10_ transformed before mean values were calculated.

#### 4.3.2. Plant Growth

*A. thaliana* Col0 seeds were surface sterilised by adding 1 mL 70% EtOH to ≈50 seeds. The seeds were incubated under constant agitation for 2 min, before they were collected by centrifugation at 1500× *g* for 1 min. The supernatant was discarded, and 1 mL sterilisation solution was added (1.17 mL bleach (12% NaOCl), 0.83 mL ddH_2_O, 20 µL 20% Triton × 100). The seeds were then incubated under constant agitation for five minutes before they were collected by centrifugation at 1500× *g* for 1 min. To remove residual sterilisation solution, we washed the seeds five times by adding 1 mL sterile water, centrifuging them, and then discarding the supernatant, after which we added 1 mL of sterile water. For stratification, seeds were stored at 4 °C for four days.

For plant cultivation, all wells of 24-well microtiter plates were filled with 1 mL ½ strength Murashige and Skoog (MS) agar (2.2 g L^−1^ MS powder including vitamins (Duchefa, Haarlem, The Netherlands), 10 g L^−1^ sucrose, 5.5 g L^−1^ plant agar (Duchefa), pH adjusted to 5.8), after which the plates were exposed to UV light in a laminar flow for 15 min [47]. Individual stratified seeds were placed into each well of the prepared microtiter plates; then, the plate was closed using Parafilm^®^ and placed in a translucent plastic bag. Plants were then grown in a plant growth chamber (Percival, Perry, IA, USA) at long day conditions (16 h day/8 h night, 22 °C day, 18 °C night, 70% relative humidity). Plants were grown 3 to 3.5 weeks and developed between six to eight leaves before they were inoculated with bacteria.

#### 4.3.3. Plant Inoculation with Conjugation Partners and Harvest

Bacterial strains were grown overnight on LB agar plates containing appropriate antibiotics. Freshly grown colonies of each bacterial strain were harvested using an inoculation loop, and the bacteria were resuspended in 10 mL 1 × PBS, washed twice by centrifugation at 3500× *g*, and resuspended in 1 × PBS. Optical density at 600 nm was determined for the cell suspensions. For single strain growth experiments, the optical density of each strain was set to OD_600nm_ = 0.2 before 20 µL of bacterial suspension were pipetted onto the middle of individual plant rosettes. For in planta conjugation experiments, donor and recipient were mixed in 1 × PBS and 20 µL of the mixture were pipetted onto individual plants. The inoculation densities were dependent on the experiment and inoculation densities ranged from OD_600nm_ = 0.05, 0.1, 0.25, to 0.5 of donor and recipient. For experiments described in Figure 3 and Figure 4, donors and recipients were each co-inoculated at an OD_600nm_ of 0.05. For experiments described in Figure 5, donors and recipients were mixed in ratios 1:1 (OD_600nm_ 0.05/0.05), 2:1 (OD_600nm_ 0.1/0.05), 5:1 (OD_600nm_0.25/0.05), or 10:1 (OD_600nm_ 0.5/0.05). For experiments described in Figure 6, donors and recipients were mixed in ratios 1:2 (OD_600nm_ 0.05/0.1), 1:5 (OD_600nm_ 0.05/0.25), 1:10 (OD_600nm_ 0.05/0.5), 2:1 (OD_600nm_ 0.1/0.05), 5:1 (OD_600nm_ 0.25/0.05), or 10:1 (OD_600nm_ 0.5/0.05). The inoculated plants were further incubated at standard growth conditions (16 h day/8 h night, 22 °C day, 18 °C night, 70% relative humidity). Plants were harvested at different time points, and bacteria were washed off to determine the CFU of each strain and transconjugants. To that end, 3 individual plants per treatment were individually processed. Plants were harvested using sterile forceps and the roots cut from the plants on a sterile surface with a sterile scalpel. Plants were transferred to pre-weighed 2 mL tubes and their weight was determined. To dislodge bacteria from plants, we added 1 mL 1 × PBS to a tube, vortexed it for 15 s, and after 7 min of sonication vortexed it again for 15 s. A total of 100 µL of the wash was spread on M9_lactose + appropriate antibiotic_ to select for transconjugants when *Ec*S17-1 or *Pe*299R were used as donors. When *Ec*O157:H7 was used as a donor, transconjugants were selected on LB_rif + appropriate antibiotic_. To extend the range of transconjugants detection, we performed a 10-times dilution series from the leaf wash and placed 3 µL droplets on appropriate agar selective for transconjugants.

#### 4.3.4. Statistical Analysis

Data were statistically analysed using the software Prism 7 (Graphpad Software, San Diego, CA, USA). All CFU were log-transformed before plotting or statistical tests were performed. To accommodate values below the limit of detection, we added a 1 to all values. To compare the difference of the mean between treatments, we performed a one-way ANOVA using Kruskal–Wallis test with Dunn’s correction for multiple comparisons.

## Figures and Tables

**Table 1:** Strains and plasmids used in this study and their abbreviations.

**Strain** **(Notes of Important Properties)**	**Abbreviation**	**Antibiotic Resistance**	**Reference**
*E. coli* O157:H7::MRE103 Δ*stx* (grows on lactose, red fluorescent, does not produce Shiga toxin)	*Ec*O157:H7red	Rifampicin	[46]
*E. coli* O157:H7 Δ*stx* (grows on lactose, does not produce Shiga toxin)	*Ec*O157:H7	Not applicable	NCTC 12900
*E. coli* S17-1 λpir (auxotroph)	*Ec*S17-1	Streptomycin	[33]
*Pantoea eucalypti* 299R (grows slowly on lactose)	*Pe*299R	Rifampicin	[36]
**Plasmid** **(Notes of Important Properties; Size)**	**Abbreviation**	**Antibiotic Resistance**	**Reference**
pUC18T-mini-Tn7T-Gm-eYFP (mobilisable, confers yellow fluorescence; 5.9 Kbp)	pUC18	Gentamicin	[43]
pKJK5::Plac::gfp (self-transmissible, confers green fluorescence; 54 Kbp)	pKJK5	Kanamycin	[15]
RP4::Plac::gfp (self-transmissible, confers green fluorescence; 56 Kbp)	RP4	Kanamycin	[15]

## Data Availability

All data generated or analysed during this study are included in this published article (and its Supplementary Information file).

## Supplementary Materials

The following are available online at https://www.mdpi.com/article/10.3390/antibiotics10080928/s1, Figure S1: Overview of experiments.
