# Supplementary material for: Conjugation Dynamics of Self-Transmissible and Mobilisable Plasmids into E. coli O157:H7 on Arabidopsis thaliana Rosettes
Source: Antibiotics (Basel). 2021 Jul 30;10(8):928. doi: 10.3390/antibiotics10080928 (PMC8388966; doi:10.3390/antibiotics10080928)
Supplement: Supplementary file 1 [file antibiotics-10-00928-s001.zip › antibiotics-1242956-SI.pdf]

Conjugation dynamics of self-transmissible and mobilisable plasmids into *E. coli* O157:H7 on *Arabidopsis thaliana* rosettes

Mitja N.P. Remus-Emsermann, David Aicher, Cosima Pelludat, Pascal Gisler, David Drissner

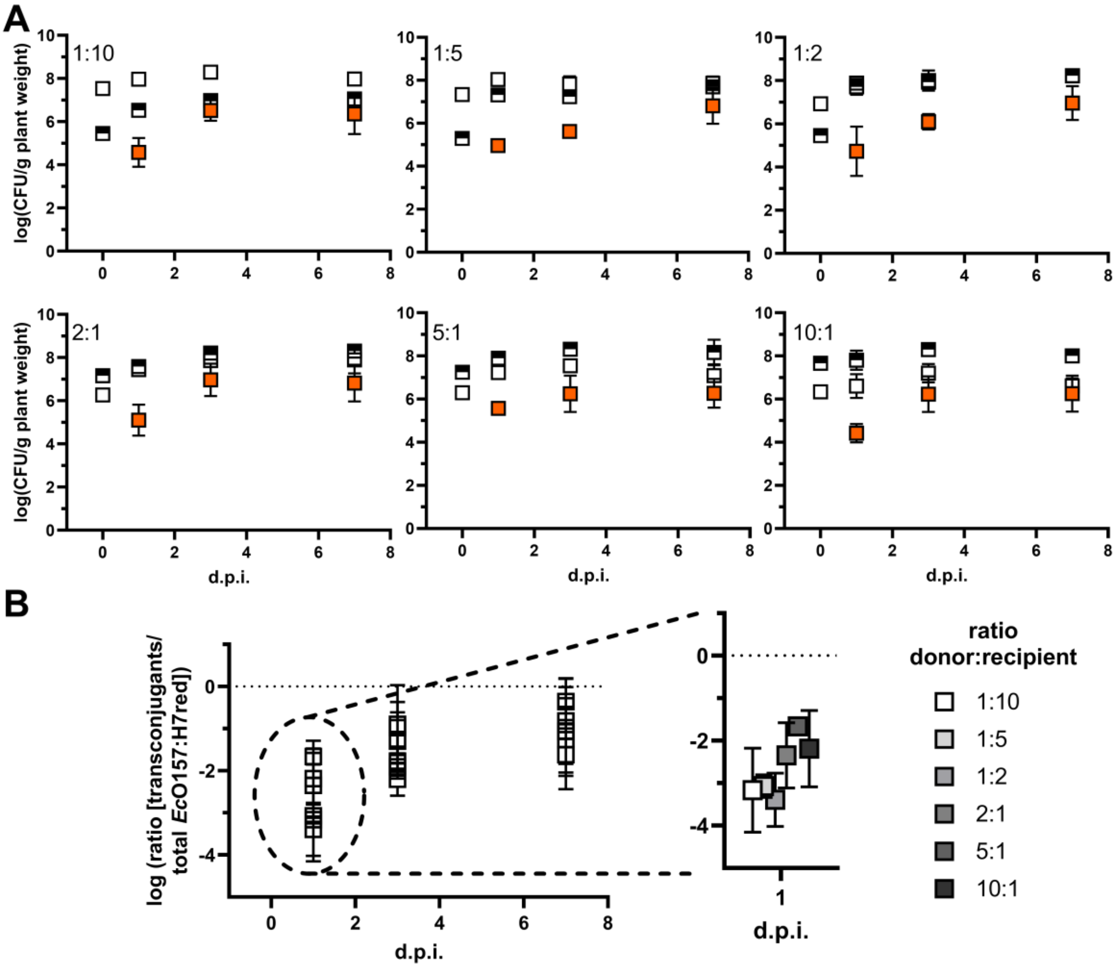

Supplementary Figure S1: Conjugation dynamics of the self-transmissible plasmid RP4 in a population of *Ec*O157:H7. **A)** Population development of *Ec*O157:H7 (RP4) donors (half filled squares), *Ec*O157:H7red recipients (open squares), and *Ec*O157:H7red transconjugants (orange filled squares) after co-inoculation of six different ratios of donor and recipient onto *A. thaliana*. **B)** Frequency of *Ec*O157:H7red transconjugants in the recipient population after co-inoculation with different densities of donor *Ec*O157:H7 (RP4) onto gnotobiotic *A. thaliana* after 1, 3 and 7 days post inoculation (d.p.i.). Error bars represent the standard deviation of the mean.
